# A Comparison of DNA Mutation and Copy Number Profiles of Primary Breast Cancers and Paired Brain Metastases for Identifying Clinically Relevant Genetic Alterations in Brain Metastases

**DOI:** 10.3390/cancers11050665

**Published:** 2019-05-13

**Authors:** Marguerite Tyran, Nadine Carbuccia, Séverine Garnier, Arnaud Guille, José Adelaïde, Pascal Finetti, Julien Touzlian, Patrice Viens, Agnès Tallet, Anthony Goncalves, Philippe Metellus, Daniel Birnbaum, Max Chaffanet, François Bertucci

**Affiliations:** 1 Laboratoire d’Oncologie Prédictive, Centre de Recherche en Cancérologie de Marseille (CRCM), Inserm, U1068, CNRS UMR7258, Aix-Marseille Université, Institut Paoli-Calmettes, F-13009 Marseille, France; tyranm@ipc.unicancer.fr (M.T.); nadine.carbuccia@inserm.fr (N.C.); garniers@ipc.unicancer.fr (S.G.); guillea@ipc.unicancer.fr (A.G.); adelaidej@ipc.unicancer.fr (J.A.); finettip@ipc.unicancer.fr (P.F.); goncalvesa@ipc.unicancer.fr (A.G.); daniel.birnbaum@inserm.fr (D.B.); chaffanetm@ipc.unicancer.fr (M.C.); 2 Département de Radiothérapie, Institut Paoli-Calmettes, 13009 Marseille, France; talleta@ipc.unicancer.fr; 3 Département d’Anatomopathologie, Institut Paoli-Calmettes, 13009 Marseille, France; Julien.touzlian@live.fr; 4 Département d’Oncologie Médicale, Institut Paoli-Calmettes, 13009 Marseille, France; viensp@ipc.unicancer.fr; 5 Faculté de Médecine, Aix-Marseille Université, 13005 Marseille, France; 6 Département de Neurochirurgie et de Neuro-oncologie, Hôpital Privé Clairval, Ramsay-Générale de Santé and Institut de Neurophysiopathologie Equipe 10, UMR0751, CNRS, 13009 Marseille, France; philippe.metellus@outlook.fr

**Keywords:** brain metastasis, breast cancer, copy number profiling, mutation, targeted therapy

## Abstract

Improving the systemic treatment of brain metastases (BM) in primary breast cancer (PBC) is impaired by the lack of genomic characterization of BM. To estimate the concordance of DNA copy-number-alterations (CNAs), mutations, and actionable genetic alterations (AGAs) between paired samples, we performed whole-genome array-comparative-genomic-hybridization, and targeted-next-generation-sequencing on 14 clinical PBC–BM pairs. We found more CNAs, more mutations, and higher tumor mutational burden, and more AGAs in BM than in PBC; 92% of the pairs harbored at least one AGA in the BM not observed in the paired PBC. This concerned various therapeutic classes, including tyrosine-kinase-receptor-inhibitors, phosphatidylinositol 3-kinase/AKT/ mammalian Target of Rapamycin (PI3K/AKT/MTOR)-inhibitors, poly ADP ribose polymerase (PARP)-inhibitors, or cyclin-dependent kinase (CDK)-inhibitors. With regards to the PARP-inhibitors, the homologous recombination defect score was positive in 79% of BM, compared to 43% of PBC, discordant in 7 out of 14 pairs, and positive in the BM in 5 out of 14 cases. CDK-inhibitors were associated with the largest percentage of discordant AGA appearing in the BM. When considering the AGA with the highest clinical-evidence level, for each sample, 50% of the pairs harbored an AGA in the BM not detected or not retained from the analysis of the paired PBC. Thus, the profiling of BM provided a more reliable opportunity, than that of PBC, for diagnostic decision-making based on genomic analysis. Patients with BM deserve an investigation of several targeted therapies.

## 1. Introduction

Breast cancer (BC) is the most frequently diagnosed cancer, worldwide, for women and the second supplier of brain metastases (BM) after lung cancer. As patients with advanced BC live longer, the incidence of BM has been increasing, and BMs occur in 15%–25% of patients. Treatment options remain limited, and in most cases, patients are excluded from clinical trials. Management requires local treatment, such as surgery or radiation therapy, or systemic therapies. Today, BM diagnosis remains associated with a poor prognosis and a poor quality of life, due to severe neurological impairments, and the median survival is ~13 months [1]. It is now recognized that certain genes increase the potential of cancer cells to metastasize to specific organs, such as bone [2], brain [3], and lung [4]. In breast cancer, the triple-negative (TN) and Human Epidermal Growth Factor Receptor-2 HER2/ERBB2-positive molecular subtypes are at a higher risk of BM [5], but the molecular bases of this tropism remain poorly understood. Ways to improve BM management include exploring the molecular mechanisms of brain invasion and extending the repertoire of potential therapeutical targets.

The BC mutatome is complex, but to date, most studies have explored primary tumors [6,7]. Metastases result from the accumulation of genomic alterations in some clones of the primary tumor, suggesting different genomic profiles between primary breast cancers (PBC) and BM. To date, very few studies have compared the genomic profiles between matched PBCs and BMs. Thus, our understanding of how a BM evolves from the primary tumor, is very limited, and the extent to which paired BMs and PBCs share genetic alterations remains unknown. Yet, the genomic-based prospective clinical trials have shown the potential of DNA-based genomic screenings applied to metastatic samples, to identify actionable genetic alterations (AGAs), for matching advanced cancers with novel targeted drugs [8,9].

In the present study, we have compared the high-throughput genomic profiles of primary tumors and matched BM from 14 patients with BC. Our aim was to measure the degree of concordance of molecular alterations between PBC and paired BM, and to compare the choices of targeted therapies, based on their respective genomic profiles, which might reveal clinically relevant AGAs enriched or restricted to the BM.

## 2. Results

### 2.1. Population

Fourteen female patients were retrospectively included. Table 1 shows their main clinico-pathological characteristics. The median-age at the time of PBC diagnosis was 51 years (range, 29 to 66). All PBCs were invasive ductal carcinomas, the pathological grade was high (grade 2–3), 29% of tumors were estrogen receptor (ER)-negative, 50% were progesterone receptor (PR)-negative, and 21% were ERBB2-positive. The PBC molecular subtypes were mainly hormone receptors HR+/HER2– (defined as ER-positive or PR-positive, and HER2–negative—57%), then HER2+ (defined as HER2–positive—21.5%), and TN (defined as ER-negative, PR-negative, and HER2–negative—21.5%). One PBC/BM pair corresponded to synchronous metastasis, whereas the other pairs were metachronous. The median-time between PBC and BM was 51 months (range, 0 to 250). During this interval, the patients received a median of four lines (range, 1 to 13) of systemic treatment—mainly chemotherapy and hormone therapy, but also targeted therapies (trastuzumab anti-HER2, bevacizumab anti-VEGF (Vascular Endothelial Growth Factor), and everolimus anti-mTOR (mammalian Target of Rapamycin)). The median overall-survival after BM occurrence was 15 months (range, 2 to 67). Two out of 13 informative pairs (15%) showed a loss of ER expression in the BM; no discordance was observed for the ERBB2 status. The molecular subtype was discordant for the two pairs (14%)—the primary tumor was HR+/HER2–, whereas the metastasis was TN.

### 2.2. DNA Copy Number Profiles

**Figure 1:**
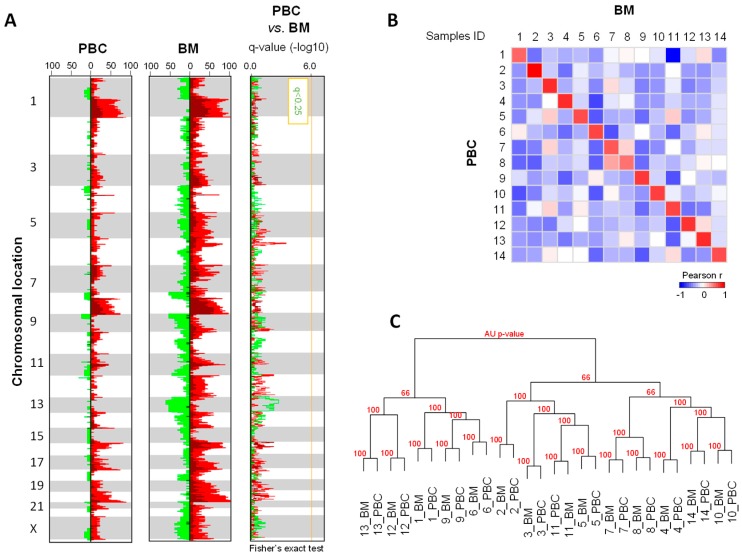
Copy-number-alteration (CNA) profiles of primary breast cancer (PBC) and brain metastases (BM)**.** (**A**) Frequency plots of genome CNAs. Frequencies (horizontal axis, 0–100%) are plotted as a function of the chromosome location (from 1pter to the top, to 22qter to the bottom), for all PBC (N = 14) and BM (N = 14). Frequencies of tumors showing CNA are color-coded (gains—light-red, amplifications—dark-red, losses—light-green, deletions—dark-green). The right plot represents the supervised analysis of CNA frequencies between PBCs and BMs. Plotted values represent the –log10 q-values of the Fisher’s exact test (red—gained/amplified regions and green—lost/deleted regions). The vertical orange line represents the significance threshold. (**B**) Correlation matrix based on the CNA profiles (log2 ratios of all probes) generated between all samples; the Pearson coefficient is color-coded according to the scale shown below the matrix. (**C**) Dendrogram of the hierarchical clustering (R-package pvclust) of the whole-genome CNAs, measured for the 28 samples (14 pairs). The AU (Approximately Unbiased) *p*-values provided by the multiscale bootstrap resampling indicate the robustness of tumor clusters; larger the *p*-values, more robust the clusters.

First, we compared the array-comparative genomic hybridization (aCGH) genomic profiles of the 14 PBCs and 14 BMs. Figure 1A represents the frequency plot of copy number alterations (CNAs), based on chromosomal locations in all samples. As expected for BC, in particular HR+/HER2–, the most frequently gained regions were located on the 1q, 8p12-qter, 11q, 16p, 17q, and 20q chromosomal arms, and the most frequently lost regions were located on the 8p and 11q chromosomal arms. No region showed a statistically different alteration frequency between the PBC and the BM. Second, a spatial analysis was done through the Genomic Identification of Significant Targets In Cancer (GISTIC) tool (Supplementary Figure S1). The common regions of CNAs between PBC and BM contained genes frequently amplified in BC—*ERBB2* (17q21), *CCND1* (11q13), and *FGFR1/ZNF703* (8p12), known to be significantly amplified in luminal B subtype [10,11,12]. However, some regions were exclusively altered in the BMs, as illustrated by the amplified 1q21.3 region containing notably the *MCL1* gene, and the lost 10q21.3 region, containing notably *CTNNA3*. Genes exclusively altered in the BM samples are listed in Supplementary Table S1.

The median percentage of probes showing CNA per sample was higher in the BMs than in the PBCs—19.3% (range, 2.2 to 25.9) versus 5.5% (range, 0.1 to 12.8; paired Mann-Whitney test, p = 0.00033). The median percentage of genes with CNA per sample was also higher in the BMs—23.5% (range, 3.2 to 33.7) versus 7% (range, 0.1 to 13.6), and the range of values was larger in the BMs than in the PBC (Supplementary Table S2). We also explored the similarities between the overall genomic profiles of the 14 PBCs and their matched BMs. We used a correlation matrix to visualize the distance of correlation (i.e., Pearson-coefficient) between all aCGH probes. Not surprisingly, each BM matched more strongly with its PBC counterpart than with all other PBCs and BMs (Figure 1B). The dendrogram of clustering obtained on all aCGH data confirmed such similarity, suggesting a clonal relationship (Figure 1C).

Finally, we focused our PBC/BM comparison on the DNA copy number of the known driver-genes of BC. Confirming the GISTIC results, *CCND1* amplification was present in 12 samples, shared between PBC and BM in five pairs (pair no. 1, 4, 10, 12, and 14), but present in the BM sample only in pairs no. 8 and no. 13, leading to a 86% concordance rate of amplified/non-amplified status. It was 65% for *MYC*, with apparition of amplification in the BM sample in four pairs (no. 9, 10, 12, and 13), thus, suggesting differences in some driver-genes. By contrast, the concordance rate was 100% for *ERBB2* and *FGFR1/ZNF703*.

### 2.3. DNA Mutational Profiles

With an average sequencing depth of 742×, we identified a total of 793 mutations. Among them, 478 different mutations, including 413 single-nucleotide-variants ((SNVs)—non-synonymous, stop-gains), 62 indels, and 3 splice-site mutations, were retained as putative somatic alterations within the 28 samples. They involved 182 different genes (detailed results in Supplementary Table S3). All samples had at least one mutation. The median number of mutations per sample was 15.5 (range, 5 to 39) for all samples, 12 (range, 5 to 39) for PBCs, and 17.5 (range, 8 to 38) for BMs, without any significant difference (Wilcoxon rank test, *p* = 0.17). The tumor mutational burden (TMB) was also higher in the BM (10.2 mutations per Megabase (Mb); range, 2.9 to 22.7), than in the PBCs (7; range, 4.6 to 22.1), but here too the difference was not significant (Student’s *t*-test, *p* = 0.198). There was also no significant difference between the PBCs and the BMs regarding the type of mutations—193 SNVs for PBCs versus 220 for BMs, and 26 indels for PBCs versus 36 for BMs (Fisher’s exact test *p* = 0.463). The similarity between each PBC and its matched BM was also measured by the correlation of the variant allele frequency (VAF) for each detected mutation—each BM strongly matched with its PBC, suggesting a strong similarity (Supplementary Figure S2A). However, some variants presented wide differences in the VAF between the PBC and the BM in the same pair (e.g., the H1047R variants of *PIK3CA*). The concordance matrix also revealed strong overall similarity between the paired BMs and the PBCs (Supplementary Figure S2B). The overall concordance rate between mutations carried by PBCs and BMs was 72% (Fisher’s exact test, *p* = 0.07), with 343 shared mutations and 135 unshared mutations (Table 2), suggesting roughly similar overall mutational profiles.

**Figure 2:**
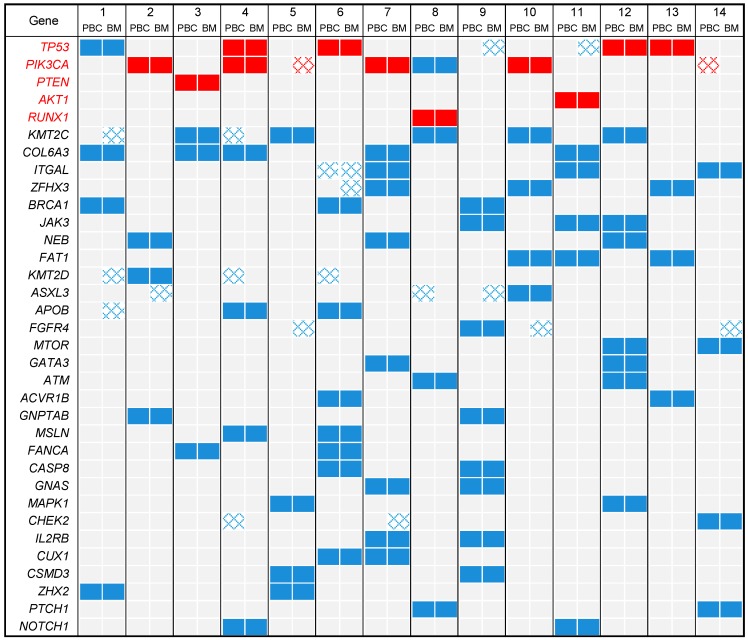
Distribution of mutations in all samples. The mutations present in at least 4 out of the 28 samples are shown, as well as all recurrent mutations. Genes are ordered from top to bottom by decreasing frequency of mutations. The genes in red present the recurrent mutations. Samples are ordered by patient number. Recurrent mutations are in red and non-recurrent are in blue. The checkerboard pattern indicates the discordant mutations between primary breast cancer (PBC) and paired brain metastasis (BM).

Figure 2 shows the distribution of all mutations present in at least four out of 28 samples, and of all recurrent mutations. Among the 478 different mutations, 25 were defined as “recurrent” and 453 as “non-recurrent”, with a uniform distribution between PBCs and BMs (*p* = 0.839). The thirteen “recurrent” variants concerned five genes—*TP53*, *PIK3CA*, *AKT1*, *PTEN*, and *RUNX1* (Supplementary Table S4). As expected for the BC samples, the most frequently mutated genes were *TP53* and *PIK3CA* [6,7]. The concordance rate was higher for the recurrent mutations (22/25; 88%) than for non-recurrent mutations (320/453; 71%, Table 2). In total, 22 out of the 28 samples had at least one recurrent mutation. Among the five genes with recurrent mutation, *PIK3CA* was the most frequently altered gene (10 samples) and the one with the largest number of different variants (5 variants). Three *PIK3CA* exon 9 recurrent hotspot mutations showed discordance in a pair—in pair n° 14, the Q546E variant was only identified in the PBC; in pair n° 5, the E545K variant was only identified in the BM; and in pair n° 7, while both PBC and BM shared the most common H1047R variant, an E542K variant was additionally identified in the BM. HR+/HER2– patients 5 and 7 had received chemotherapy and hormone therapy before the surgery for BM and patient 5 had also received bevacizumab. For *TP53*, four “recurrent” variants were found in eight samples, and each variant was identical in the PBC and its BM. A total of 12 of the 14 pairs (86%) showed concordance regarding the occurrence of those recurrent mutations, but 14% were discordant.

### 2.4. Profile of Actionable Genetic Alterations

Despite an overall similarity in terms of CNAs and mutations between PBC and BM, we observed divergence for some recurrent and clinically relevant AGAs of BC, suggesting that additional potential actionable targets might be detected, solely from the profiling of BM. According to the algorithm of Perera–Bel et al. [13], a total of 99 different AGAs (17 amplifications, 4 deletions, 81 mutations) were retained, concerning 59 genes (21 amplified or deleted, 42 mutated), with evidence level ranging from A1 to B3 (Supplementary Table S5). All samples harbored at least one AGA. In our series, these alterations were observed 192 times—132 times in both PBC and paired BM, 11 times in the PBC only, and 49 times in the BM only, suggesting more AGAs in BM. The median number of AGAs was 6 (range, 2 to 15) in the whole series, but was significantly higher in the BMs (8; range, 3 to 15) than in the PBCs (5; range, 2 to 14; Wilcoxon rank test, *p* = 0.006). The median percentage of shared AGAs, between BMs and PBCs was 50% (range, 0 to 100). All but one pair (92%) harbored at least one of these AGAs in the BM that was not observed in the paired PBC. The AGAs observed 49 times in the BM, only included 41 different alterations, corresponding to 31 out of 59 genes—they are written in red in the second and third columns of Supplementary Table S5.

**Figure 3:**
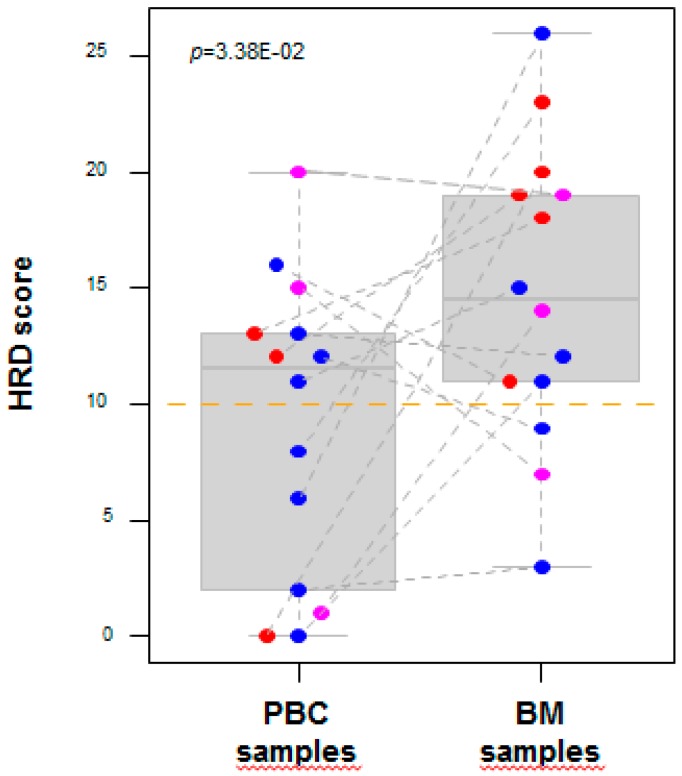
Homologous Recombination Deficiency (HRD) scores of the brain metastases (BM) and primary breast cancer (PBC) samples. Distribution of the HRD scores in all samples. The corresponding PBC–BM pairs are connected by thin dashed lines. The scores were higher in the BM samples compared to the PBC samples. The *p*-value is for the paired Student’s *t*-test.

The 99 AGAs predicted sensitivity to different drugs, mainly represented by tyrosine kinase receptors (TKR)-inhibitors and the poly-ADP-ribose-polymerase (PARP)-inhibitors. Regarding the TKR-inhibitors, 16 different AGAs were observed—the most frequently altered genes in BMs, overall, were *FGF3* and *FGF4*, and the most frequently altered genes only in BM were *EGFR*, *FGF3*, and *FGF4* (2/14 BM for each gene). This diagnostic class was the class associated with the largest total number of occurrences (N = 13) of discordant AGAs, and notably of those corresponding to the apparition in the BM sample. For the PARP-inhibitors, 16 different AGAs were observed; the most frequently altered gene in BMs, overall, was *BRCA1*, and the most frequently altered genes only in BM, were *BRCA2*, *CHEK2*, *PALB2*, and *STAG2* (1/14 BM for each gene). Interestingly, the Homologous Recombination Deficiency (HRD) score was higher in the BM samples than in the PBC samples (*p* = 3.38E-02, paired Student’s *t*-test; Figure 3). Eleven out of 14 BM samples (79%) were HRD-positive (score > 10), compared to 43% of the PBC samples (6 out of 14). The 11 BM samples included 5 TN and 2 HER+, but also 4 HR+/HER2–subtypes. Of note, 7 out of 14 pairs displayed a modification of the HRD score between the PBC and the BM—from negative to positive in five (36%) cases (3 HR+/HER2–, 1 TN, and 1 HER2+ PBC), and from positive to negative in two (14%) cases (1 HR+/HER2–, and 1 HER2+ PBC). Regarding the phosphatidylinositol 3-kinase/AKT/ mammalian Target Of Rapamycin (PI3K/AKT/MTOR)-inhibitors, 13 different AGAs were observed; the most frequently altered gene in the BMs, overall, and the most frequently altered gene only in BM was *PIK3CA* (4/14 BMs). Regarding the cyclin-dependent kinases (CDK)-inhibitors, 7 different AGAs were observed; the most frequently altered gene in the BMs, overall, was *CCND1*, and the most frequently altered genes only in BM, were *RB1* and *CCND1* (2/14 BMs for each gene). This class was the class associated with the largest percentage of discordant AGAs (relatively to the total number of alterations in this class), with 57% of alterations present in the BMs only, versus 0% only in the PBC. Regarding hormone therapy, the most frequently altered gene only in BM was *ESR1* (2/14 BMs). Among the genes with other AGAs, the most frequently altered genes only in BM were *MYC* (4/14 BMs), and *TP53*, and *MDM4* (3/14 BMs).

Thus, many additional therapeutic targets were identified in the BM samples. From a clinical practical point-of-view, we retained for each sample the AGAs with the highest evidence level (A1 > B1 > A2 > B2 > A3 > B3), which would guide the “first-line genomics-based” therapeutic choice and compared it between BM and paired PBC. As shown in Table 3, 7 out of 14 pairs (50%) had at least one such alteration (evidence level ranging from A1 to B2) in the BM, which differed from the one retained in the paired PBC, suggesting that in clinical practice, the profiling of BM would suggest a different targeted therapy than that of PBC, in many cases. These “additional targets” were *EGFR* mutation for patient 4 and amplification for patient 11, *FGF3* and *FGF4* amplifications for patients 8 and 12, *PIK3CA* mutations for patients 5 and 7, *MTOR* mutation for patient 14, *ESR1* mutations for patients 7 and 8, and *CHEK2* mutation for patient 14.

## 3. Discussion

Our aim was to estimate the degree of concordance of CNAs, mutational profiles, and AGAs, between PBCs and paired BMs. We showed that, overall, the molecular profiles were concordant. However, there were differences for genes that were recurrently altered and clinically relevant in BC, or other cancers, including many therapeutic targets. Ninety-two percent of pairs harbored at least one AGA in the BM that was not observed in the paired PBC. In 50% of cases, the highest clinical-evidence level AGA identified in the BM would have suggested a “first-line genomics-based” therapeutic choice, different from the one identified in the paired PBC.

Despite the multiple systemic treatments received by our patients, between the removals of the PBC and the paired BM, which were separated by a median time-interval of 51 months, and despite the known genetic instability of cancer cells, we found a high-level of global concordance between the primary and brain secondary tumors. Similarity of DNA copy-number profiles was suggested by similar frequency plots and by clustering and a correlation matrix, in which, each BM correlated more strongly with its paired PBC than with other samples. The only statistical difference was quantitative—BM presented a higher average number of probes or genes with CNA than the PBC samples. We did not find this observation in our previous study [14], in which the matched metastases were from the non-cerebral sites. At the gene level, the concordance rate was variable among the known BC driver-genes—100% for *FGFR1/ZNF703* and *ERRB2*, 86% for *CCND1* (indicating possible differences in the sensitivity to CDK-inhibitors between PBC and the paired BM), and 65% for *MYC*.

The similarity of global mutational profiles between PBC and the paired BM was substantiated by the identical number of variants and mutational load per sample, by identical type of mutations (SNVs, indels), by the correlation matrix based on VAF, and by an analysis of concordance of the detected variants. The degree of concordance was 72% when considering all variants, and higher for the “recurrent’ variants (88%), which might involve potential driver-genes. Concerning the “recurrent” variants (five genes), the discrepancies observed in our study concerned *PIK3CA*, only, which belongs to a list of genes with known frequent clonal divergences [15]. The presence of mutation in the PBC of pair n°14 but not in the BM suggests that the metastasis might have branched off before the acquisition of the mutation by the PBC, whereas the presence of a novel mutation in the BM of pairs no. 5 and no. 7, might be explained by an accumulation of new mutations over time. Although activation of the PI3K signaling is associated with the poor-prognosis luminal B subtype, and is accompanied by the development of endocrine therapy resistance, and although inhibition of the PI3K pathway signaling in endocrine-resistant breast cancer improves the response to hormone therapy [16], the acquisition of *PIK3CA* mutations did not appear to be responsible for the development of endocrine resistance, per se [17]. Thus, it was not clear whether the apparition of these exon 9 mutations was due to resistance to hormone therapy or to disease progression. Concerning “non-recurrent” variants, a mutation in *ESR1* was discovered in the BM of patient no. 7 (E380Q variant) and patient no. 8 (Y537C variant). These patients had a very long interval between the PBC and BM (185 and 250 months, respectively), along which they received several years of hormonal-therapy that had led to the acquisition of the *ESR1* mutation conferring therapeutic resistance [18].

Due to the rarity of the available clinical BM samples, most research on BM from BC had historically focused on gene expression analysis of clinically annotated PBCs, and functional analysis of preclinical models [3,19,20]; and several candidate genes for BC metastatis to the brain have been proposed [3,21,22,23,24,25,26,27,28,29,30,31,32,33]. Recent studies have profiled small series of BM samples, without matched PBC [24] or compared the molecular profiles of BM and matched PBC, but mainly at the transcriptional level [23,25]. In the literature, only six studies focused their high-throughput DNA analyses on matched PBC and BM, but in small series, and none combined aCGH and Next-Generation Sequencing (NGS) [21,22,23,33,34,35]. Somatic mutation profiling of 19 genes was applied to 12 matched pairs [23]. Except for one *EGFR* mutation, similar somatic mutations for *NRAS* and *PIK3CA* were observed in the BMs and in the PBCs. The sequences of a 50-gene panel were established in 60 samples, including 15 matched pairs [21] (43% were TN). The most common mutations in the 42 BMs affected were in *TP53*, *RB1*, *PIK3CA*, *KIT*, and *MLH1*. In agreement with our results, no significant difference in mutation profiles was found between the two paired groups, even if *TP53* mutation was more frequent in the BMs than in the PBCs (59% versus 39%, respectively). A gene copy number analysis by aCGH was done on 10 BMs from BC, including only four that were compared to their paired PBC [33]. No statistical analysis could be applied to the comparison of paired samples, but CNAs of therapeutic targets were identified in BMs only. More recently, the NGS profile of 254 genes was established in matched pairs of BM and PBC in six patients with HER2+ breast cancer [34]: changes in clonal composition and acquisition of mutations private to or enriched in the BM affected clinically actionable cancer genes, including *ATR*, *BRAF*, *FGFR2*, *MAP2K4*, *PIK3CA*, *RAF1*, and *TP53*. Of note, four out of the six cases harbored likely pathogenic *TP53* mutations, private or enriched in the BM, and associated with the Loss of Heterozygosity (LOH) of the wild-type allele. Four cases (67%) harbored at least one diagnostic AGA private to or enriched in the BM. By reanalyzing the 21 Brastianos’ PBC/BM pairs and by analyzing the 17 new pairs using Single-Nucleotide Polymorphism (SNP)-array and NGS of 111 genes, a previous study [35] demonstrated that the various DNA aberration-based HRD measures were significantly higher in BM than in their PBC counterparts. Such an increase had been previously suggested in a publication showing that a *BRCA1* deficient-like gene expression signature was higher in breast cancer BM [36]. Our analysis confirmed such an enrichment in HRD in BM, and notably in HR+/HER2– samples. The largest study [22] of whole-exome sequencing analyzed 86 matched primary tumors (lung, breast, and others), BMs and normal tissues, including 21 PBCs. In all clonally-related cancer samples, branched evolution was observed, i.e., all metastatic and primary sites shared a common ancestor yet continued to evolve independently. In contrast, there was strong genetic homogeneity between spatially and temporally separated BMs, but strong difference between BMs and distal extra-cranial metastases. In 53% of the 86 pairs, potentially clinically informative alterations were found in the BMs and not detected in the matched primary tumor. Unfortunately, such a figure was not provided for the PBC group specifically. Similarly, in our series, and when considering only the AGAs with the highest clinical evidence level, 50% of BMs showed additional therapeutical AGA not present in the paired PBC, and this figure was much higher (92%) when considering all evidence level alterations. Such AGAs observed in the paired BM might only represent the alterations of resistance acquired during the systemic therapy, delivered before the brain relapse. For example, among the AGAs with highest clinical evidence level, this was very likely the case for *ESR1* mutations after hormone therapy [18], and a few studies suggest that it might be the case for *PIK3CA* mutations after hormone therapy [16,17] and/or chemotherapy [37], for *EGFR* mutation and amplification after hormone therapy [38], and for *FGF3*/*FGF4* amplification after hormone therapy [39,40,41] and after trastuzumab [42]. Further analyses are required to test such hypotheses. Similarly, the appearance of the *MTOR* mutation in the patient 14, who was previously treated with everolimus, is intriguing and deserves investigation.

Despite global genetic similarity, we showed that important differences were observed between PBCs and BMs, which might have a therapeutic impact. Using a recently published automated model, we showed that more AGAs were present in the BMs than in the paired PBCs. Such additional alterations might confer sensitivity to different therapeutic classes, including Tyrosine Kinase Receptors (TKR)-inhibitors, PARP-inhibitors, CDK-inhibitors, and hormone therapy. The CDK-inhibitors were the therapeutic class associated with the largest percentage of discordant alterations (relative to the total number of alterations in this class). The TKR-inhibitors were the class associated with the largest total number of occurrences of discordant alterations, and notably of those corresponding to apparition in the BM sample. Regarding the PARP-inhibitors, several data suggest higher efficiency for *BRCA* mutation or a high HRD score [43]. This concept called “synthetic lethality” is due to the essential role of PARP1 in the base excision repair of DNA single-strand breaks (SSBs). In its absence, DNA SSBs accumulate and degenerate to DNA double-strand breaks, which are not appropriately repaired in the case of HRD. Based on the AGAs, four patients displayed apparition in the BM sample of alteration in genes involved in homologous recombination (*BRCA2*, *CHEK2*, *PALB2*, and *STAG2*). Based on the HRD score, five patients displayed apparition of a positive score in the BM. All these patients might, thus, become candidates to PARP-inhibitors, based on the profiling of the BM. Of note, 79% of BM samples were HRD-positive (score >10), and this concerned all molecular subtypes, including the HR+/HER2– subtype. Manually, we identified other potential AGAs present only in the BMs, such as the *MCL1* amplification known to be associated with sensitivity to CDK-inhibitors [44], or *CD274* amplification, supporting a sensitivity for Programmed Death-Ligand 1 (PDL1) inhibitors [45]. Such discrepancy in term of therapeutic targets, and notably the presence of targetable alterations in cancer subclones specific to BM, provides strong opportunity for novel targeted therapeutic strategies that might affect the overall survival, since more than 50% of patients with BM die of intra-cranial progression. It might also explain the discordance of therapeutic response in some cases between PBC, extra-cranial metastases and BM, which has often been attributed to the low penetration of systemic drugs across the blood–brain barrier, but there is a growing body of evidence that targeted therapies can traverse this barrier and result in clinical benefit for patients with BM [46,47,48,49]. Many potential targets for patients are described for patients with BC and BM, and several clinical trials are ongoing [50,51]. Another interesting issue might be to assess the concordance of molecular alterations between BM and PBC, by molecular subtype or by age group, but the small series size precludes any reliable statistical analysis. For example, we compared the concordance rate by age group (‘younger’: ≤50 years and ‘older’: >50). Regarding the two known driver genes that showed amplification discordance between BM and PBC (*CCND1* and *MYC*), the discordance concerned the two age groups. Regarding the mutations, the concordance rate was higher in the younger group than in the older group, for all mutations (79% versus 66%, *p* = 0.002) and for the non-recurrent mutations (78% versus 65%, *p* = 0.003), but was not different for the recurrent mutations (92% versus 80%, *p* = 0.6). Regarding the 192 AGAs, the median percentage of shared AGAs between BMs and PBCs was not different (*p* = 0.3) between the younger (43%; range, 33 to 55) and the older (59%; range, 0 to 100) groups. Regarding the highest evidence-level AGAs, the seven patients with discordant (AGAs) present only in the BM were evenly distributed in both groups.

To our knowledge, this study was the first one presenting the results of both aCGH and targeted NGS, on the matched pairs of PBC and BM. Beside its retrospective nature, it displayed a few other limitations. (i) Small number of cases, even if it was the third largest study in term of genome profiling of PBC/BM pairs after Lee’s [21] (15 pairs) and Brastianos’s series [22] (21 pairs), and such matched pairs were very rare due to the difficulty of collecting BM. (ii) Heterogeneity of the population in terms of molecular subtypes, and the statistical impossibility of analysis per subtype, given the small number of cases. (iii) Delivery of different systemic treatments before the metastatic progression. (iv) Relatively small number of genes analyzed, when compared with the whole-exome or whole-genome sequencing. (v) Absence of functional analyses to demonstrate the role of identified genetic alterations in the formation of BM and the therapeutic interest to target them. (vi) Limitation and evolution of the currently available databases of actionable alterations, such as the algorithm we used. It is also not clear whether the enrichment observed for certain alterations was common to other metastatic sites or were specific to the brain site. Pre-clinical analyses and prospective clinical studies are required to answer these crucial questions. Finally, the comparison between the PBC and BM should also include, notably, proteomics and phospho-proteomics analyses. Nevertheless, today, aCGH and NGS represent the backbone of precision medicine.

## 4. Materials and Methods

### 4.1. Patients and Samples Selection

All samples were collected from patients who underwent surgical resection of PBC at the Institut Paoli-Calmettes (IPC; Marseille, France) and of BM at the Clairval Hospital (Marseille, France). After surgery, samples had been respectively stored in the IPC tumor bank (AC-2018-1905) and the Assistance Publique-Hôpitaux de Marseille (AP-HM) tumor bank (AC-2013-1786) in Marseille France. The patients were selected by interrogating the respective institutional databases. Other selection criteria included—available frozen sample of both operated PBC and paired BM, tumor cellularity assessment by pathologists to guide DNA extraction (>50%), available clinico-pathological data, and written informed patient’s consent. Samples were frozen in liquid nitrogen, within 30 minutes of surgery. The extraction was guided by the pathologist who circled the area in which the tumor cellularity was evaluated. Tumor-DNA was extracted, as previously described [14], and quality was controlled on polyacrylamide gel electrophoresis; DNA-concentration was assessed by using Qubit dsDNA-BR-Assay (ThermoFisher Scientific, Walthan, MA, USA). Out of the 15 matched pairs identified (15 patients), 14 with good DNA quality were retained for analysis. The study (No. 13-002) was approved by our institutional review board (Comité d’Orientation Stratégique IPC) on February 23, 2016.

### 4.2. DNA Copy Number Profiling

For each sample, the genomic profile was established by using array-comparative genomic hybridization (aCGH) onto high-resolution 4 × 180K CGH-microarrays (SurePrint G3-Human CGH-Microarray, Agilent Technologies, Massy, France). Human female-DNA was used as reference (G152A, Promega, Madison, WI, USA). Both experimental and analytic methods have been previously described [10,14]. All probes for aCGH were mapped according to the Genome Reference Consortium Human Build 37 (CGCh37/Hg19) (https://www.ncbi.nlm.nih.gov/assembly/GCF_000001405.13/). We used two different threshold values (log2 ratio > |0.5| and |1|) to distinguish low (gain/loss) from high (amplification/deletion) level copy-number-alterations (CNA), respectively [10]. To identify the overall altered regions, we used the GISTIC2.0 algorithm [52], calculated by multiple random iterations, with a corrected threshold probability (q < 0.25) to define a statistically relevant region. The homologous recombination deficiency score, HRD score [53] was defined on the segmented data processed with circular binary segmentation [54]. This HRD score was based on the number of loss of heterozygosity (LOH) regions of intermediate size. It was defined in a study of 152 ovarian carcinomas that examined the association between HRD and genomic patterns of LOH. Its robustness was validated in two independent data sets of ovarian carcinomas (N = 488), as well as 57 breast and pancreatic cancer cell lines. This HRD score was capable of detecting HRD, regardless of etiology or mechanism. Data will be available after acceptance of the paper (Array_Express accession number: E-MTAB-7118).

### 4.3. Mutational Profiling

Targeted next-generation-sequencing (tNGS) was applied to a custom-made panel of 494 “cancer-associated” and “actionable” genes (CCP-V8 panel, Supplementary Table S1). For each of the 28 samples, the DNA-libraries of all coding exons and intron-exon boundaries of all genes were prepared using the HaloPlex Target-Enrichment-System (Agilent, Santa Clara, CA, USA), as previously described [55]. Sequencing was done using the 2 × 150-bp paired-end technology on the Illumina NextSeq500 platform, according to the manufacturer’s instructions (Illumina, San Diego, CA, USA). Sequence data were aligned to the human reference-genome (UCSC hg19) and analyzed, as previously described [14,55]. A “recurrent” mutation was defined as being found more than 10 times in the Catalogue of Somatic Mutations in Cancer (COSMIC V68) database (http://cancer.sanger.ac.uk/cosmic); the other variants were defined as “non-recurrent”. The tumor mutational burden (TMB), a potential predictor of response to immunotherapy in diverse cancers, was defined as the number of non-synonymous somatic, coding, base substitution, and indel mutations per megabase of genome examined. It was calculated by dividing the total number of mutations by the size of the coding region of the targeted genes territory associated with the comprehensive cancer panel (CCP-V8) panel [56]. Computing resources for this study was provided by the computing facilities DISC (Datacenter IT and Scientific Computing) of the Centre de Recherche en Cancérologie de Marseille. Data will be available after acceptance of the paper (EGA accession number: EGAS00001003173).

### 4.4. Definition of Actionable Genetic Alterations

To define the landscape of AGAs, we applied the algorithm developed by Perera-Bel et al. [13], which matches patient-specific genomic alterations to treatment options. This model is based upon public knowledge of somatic variants with predictive evidence on drug response. It is based upon several public data, including Gene-Drug-Knowledge-database (GDKD), Clinical-Interpretation-of-Variants-in-Cancer (CIViC), and Tumor-Alterations-Relevant-for- Genomics -driven-Therapy (TARGET). The molecular alterations of the 312 actionable genes are classified into a six-level system to rank the associations, according to their evidence. The system uses two axes representing the strength of clinical evidence (axis 1/2/3) and the cancer-type (axis A/B). Levels A and B mean evidence in the same cancer-type and in any other cancer-type, respectively. Level-1 means supported by drug approval organizations/clinical guidelines, level-2 contains clinical evidence, in which late clinical trials are ranked higher, followed by early clinical trials and case reports, and level-3 consists of preclinical evidence. The highest level is A1, then B1, then A2, then B2, then A3, then B3. In our analysis, we considered only alterations noted as associated with “response” or “sensitivity” to drugs, by excluding those associated with “no response” or “resistance”.

### 4.5. Statistical Analysis

The comparison of the frequency of probes with CNA between PBC and BM was done using Fisher’s exact-test (discrete variables) and the Mann-Whitney test (continuous variables). Analysis of the correlation of CNA data (log2-ratio of all probes) of each BM with all PBC was done using the Pearson-coefficient. The hierarchical clustering of whole-genome CNA data used the R-package pvclust (Kyoto University, Japon) [57] with the following parameters—Ward’s agglomerative method, Pearson-correlation, and 100 bootstrap replications, to assess the robustness of clusters. Regarding the mutational profiles, the comparison of the number of mutations per sample was done using the Wilcoxon-rank test, and the comparison of mutational load, using the Student’s t-test. The similarity of samples was measured using the Pearson-correlation, based on the frequency of allelic variants (VAF) for all detected variants of each metastasis, with all PBC. Concordance analysis was done as described in [58] and based on the –log10 p-values of the Fisher’s exact-test of a 2-by-2 contingency table. The significance of the *p*-values threshold was set at 5% and the analyses used the R-software (Vienna, Austria) (version 2.15.2).

## 5. Conclusions

We have shown overall concordance between BM and paired PBC. However, we found more CNAs (significant difference) and more mutations (not significant) in the BM samples, and above all, important genetic differences that might be clinically interesting for precision medicine, with more AGAs in BMs than in PBCs. However, today the therapeutic decisions for patients with BM remain, based on biomarkers assessed in the primary tumor. Since the main cause of death in patients is the evolution of BM and many targeted therapies are currently being tested in clinical trials, including patients with BM, the therapeutic targeting of such discordant alterations might affect the overall survival and deserves further investigation, at least in patients with a molecular target identified in the BM. Currently, American Society of Clinical Oncology (ASCO) guidelines recommend a biopsy for retesting ER, PR, and ERBB2 in BC patients with accessible metastases [59]. Despite their respective limitations of the corresponding published studies, and based on Brastianos’ and De Matteos-Arruda’s results [22,34], and ours, it seems clear that craniotomy operative specimen provides an immediate and ideal opportunity for decision-making, based on genomic analysis in patients with BM, which is more reliable than PBC, and regional and distant extra-cranial metastases. Even if many patients have a resection of BM as part of the clinical care, identification of surrogate markers of BM actionable genetic alterations in more accessible clinical tissues, is warranted. Moreover, there is ongoing research on using tumor-derived DNA isolated from cerebrospinal-fluid or blood. Beside their clinical interest, such samples will be crucial to help understand the BM process and to collect precious data for future research.

## Figures and Tables

**Table 1:** Clinicopathological characteristics of patients and samples.

Samples	Age (Years)	Time Before BM Occurrence (Months)	No. and Type of Systemic Therapies Before BM Occurrence	Tumor Cellularity (%)	ER Status	PR Status	ERBB2 Status	Molecular Subtype	Survival After BM Occurrence (Months)
PBC1	51		4 (CT, beva)	100	NEG	NEG	NEG	TN	
BM1		54		100	NEG	NEG	NEG	TN	11
PBC2	66		4 (CT, trastu)	100	NEG	NEG	POS	HER2+	
BM2		53		100	NEG	POS	POS	HER2+	18
PBC3	62		5 (CT, HT)	100	POS	POS	NEG	HR+/HER2–	
BM3		49		70	POS	NEG	NEG	HR+/HER2–	30
PBC4	61		2 (CT, HT)	100	POS	POS	NEG	HR+/HER2–	
BM4		28		80	POS	POS	NEG	HR+/HER2–	2
PBC5	51		5 (CT, HT, beva)	100	POS	NEG	NEG	HR+/HER2–	
BM5		173		80	POS	NEG	NEG	HR+/HER2–	24
PBC6	43		2 (CT)	80	NEG	NEG	NEG	TN	
BM6		22		70	NEG	NEG	NEG	TN	7
PBC7	39		6 (CT, HT)	100	POS	POS	NEG	HR+/HER2–	
BM7		185		60	POS	POS	NEG	HR+/HER2–	22
PBC8	43		5 (CT, HT, trastu)	100	POS	POS	POS	HER2+	
BM8		250		60	POS	POS	POS	HER2+	56
PBC9	29		1 (CT)	100	NEG	NEG	NEG	TN	
BM9		43		60	NEG	NEG	NEG	TN	67
PBC10	58		2 (CT, HT)	100	POS	NEG	NEG	HR+/HER2–	
BM10		0		100	NEG	NEG	NEG	TN	12
PBC11	40		13 (CT, HT)	100	POS	POS	NEG	HR+/HER2–	
BM11		106		100	NR	NR	NEG	HR+/HER2–	6
PBC12	41		6 (CT, HT, beva)	100	POS	NEG	NEG	HR+/HER2–	
BM12		75		100	POS	POS	NEG	HR+/HER2–	11
PBC13	61		2 (CT, HT, trastu)	100	POS	POS	POS	HER2+	
BM13		19		100	POS	POS	POS	HER2+	26
PBC14	65		3 (CT, HT, éverolimus)	75	POS	POS	NEG	HR+/HER2–	
BM14		32		50	NEG	NEG	NEG	TN	4

* CT—chemotherapy; HT—hormone therapy; trastu—trastuzumab; beva—bevacizumab; ER-estrogene receptor; PR-progesterone receptor; BM-brain metastasis; PBC1-primary breast cancer of patient 1; NEG-negative; POS-positive; TN-triple-negative; HR-hormone receptor; HER2-Human Epidermal Growth Factor Receptor-2

**Table 2:** Concordance between PBC and paired BM for all detected mutations.

	Total Number of Mutations	Number of Shared Mutations	Number of Unshared Mutations	Concordance Rate between Paired PBC and BM
All mutations	478	343	135	72%
Recurrent mutations	25	22	3	88%
Non-recurrent mutations	453	320	133	71%

PBC: primary breast cancer, BM: brain metastasis.

**Table 3:** List of the highest evidence level actionable genetic alterations for each sample.

Therapeutic Class	Gene Symbol	Alteration	Highest Evidence Level	Pt 1		Pt 2		Pt 3		Pt 4		Pt 5		Pt 6		Pt 7		Pt 8		Pt 9		Pt 10		Pt 11		Pt 12		Pt 13		Pt 14	
				PBC	BM	PBC	BM	PBC	BM	PBC	BM	PBC	BM	PBC	BM	PBC	BM	PBC	BM	PBC	BM	PBC	BM	PBC	BM	PBC	BM	PBC	BM	PBC	BM
**PIK3/AKT/MTOR inhibitors**	*AKT1*	E17K	B2																												
		D44N	B2																												
	*MTOR*	A607V	B2																												
	*PIK3CA*	E542K H1047R	A2																												
		E545K	A2																												
		H1047R	A2																												
		Q546E	A2																												
	*NF1*	D176E	B2																												
	*PTEN*	Q171X	A2																												
PARP inhibitors	*ATM*	G2023R	B2																												
	*BRCA1*	E1282fs 1280-1281del	B1																												
		E881X	B1																												
		Q1811R	B1																												
	*BRCA2*	Y2222C	B1																												
	*CHEK2*	T367fs	B2																												
TKR inhibitors	*EGFR*	amplification	B2																												
		M137I	B1																												
	*ERBB2*	amplification	A1																												
	*RET*	L389F	B1																												
	*FGF3*	amplification	A2																												
	*FGF4*	amplification	A2																												
	*FGFR1*	amplification	A2																												
Hormone therapy	*ESR1*	E380Q	A2																												
		Y537C	A2																												
Epigenetic therapy	*SMARCA4*	D1607N	B2																												
Others	*TP53*	R196Q	B2																												

Orange—gene amplification, blue—non-recurrent mutations, red—recurrent mutations; checkerboard pattern—discordant mutations between matched primary breast cancers (PBC) and brain metastases (BM).

## Supplementary Materials

The following are available online at https://www.mdpi.com/2072-6694/11/5/665/s1, Figure S1: GISTIC analysis of CNA profiles; Figure S2: Concordance of variant allele frequency (VAF) and alterations based on mutational profiles; Table S1: Genes with CNA exclusively found in the BM exclusive samples; Table S2: Number of genes with CNA in PBC and matched BM; Table S3: Details of all detected mutations in all samples; Table S4: List of 13 recurrent somatic variants detected in the 28 samples; Table S5: List of all-evidence level actionable genetic alterations; Table S6: Panel of 494 “cancer-associated” genes (CCP-V8 panel).
