# Supplementary material for: A Comparison of DNA Mutation and Copy Number Profiles of Primary Breast Cancers and Paired Brain Metastases for Identifying Clinically Relevant Genetic Alterations in Brain Metastases
Source: Cancers (Basel). 2019 May 13;11(5):665. doi: 10.3390/cancers11050665 (PMC6562582; doi:10.3390/cancers11050665)
Supplement: Supplementary file 1 [file cancers-11-00665-s001.zip › Supplementary/supplementary.docx]

**
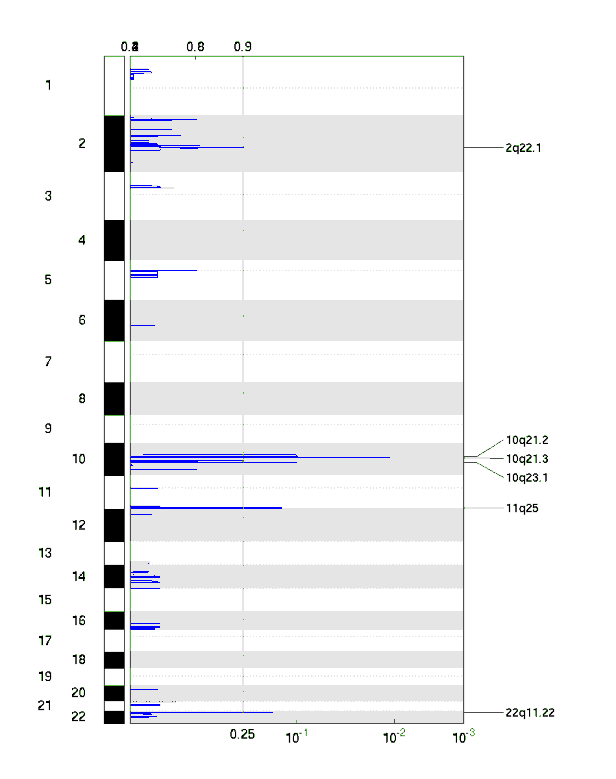

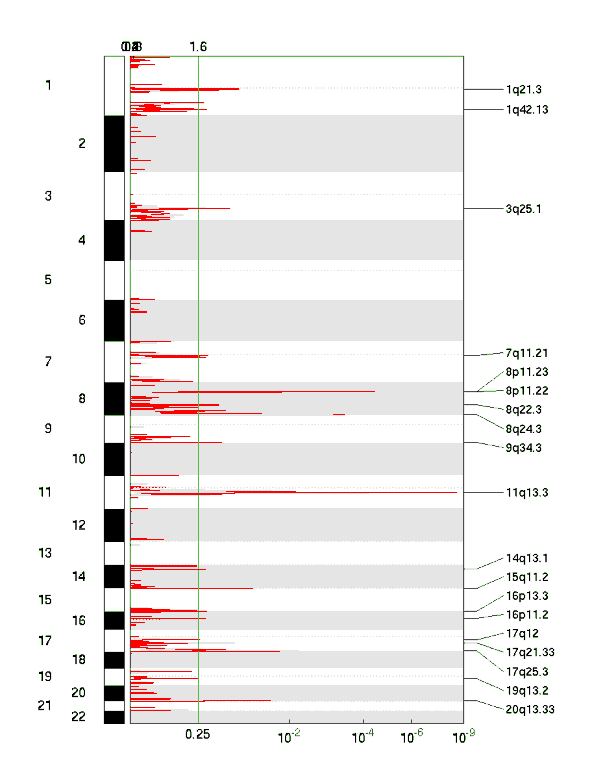

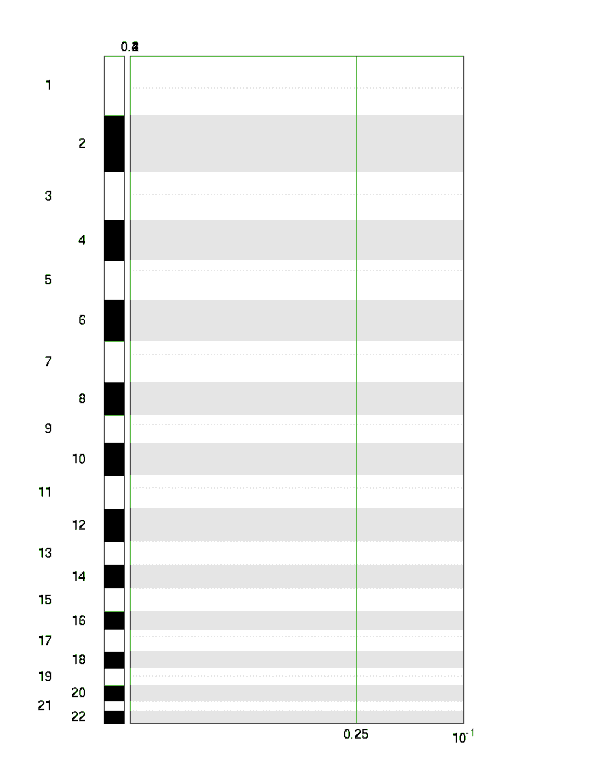
Supplementary** **Materials**

***q***

***q***

***q***

***q***

**BM**

**PBC**

***MCL1*** (1q21.3)

***CTNNA3*** (10q21.3)

***CCND1*** (11q13.3)

***ZNF703*** (8p11.23)

***ERBB2*** (17q12)


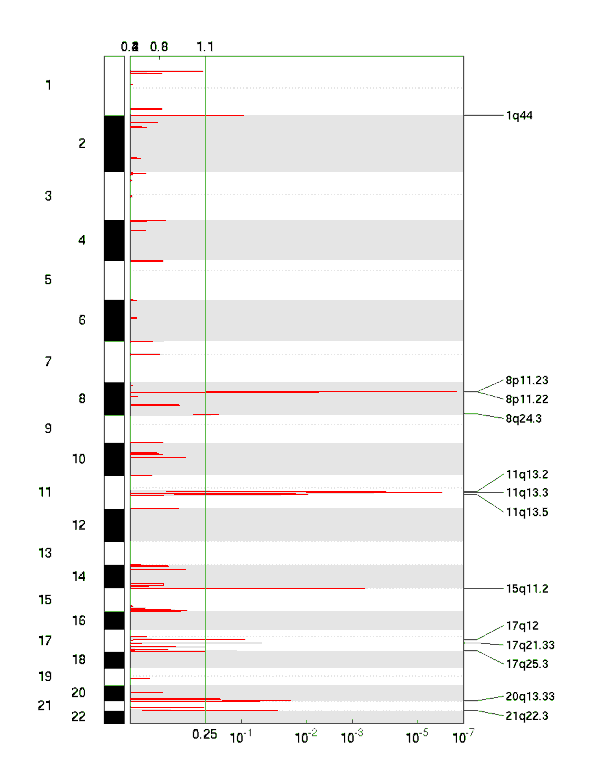


**GISTIC Deletion score**

**Chomosom location**

**Chomosom location**

**Chomosom location**

**Chomosom location**

**GISITIC Amplification score**

**Figure S1.** GISTIC analysis of CNA profiles.

1

2

3

4

5

6

7

8

9

10

11

12

13

14

14

13

12

11

10

9

8

7

6

5

4

3

2

1

**BM**

**PBC**

Samples ID


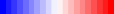


0

1

-1

Pearson r

**A**

**B**

1

2

3

4

5

6

7

8

9

10

11

12

13

14

14

13

12

11

10

9

8

7

6

5

4

3

2

1

**BM**

**PBC**

Samples ID


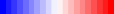


<0.05

p-value

**Figure S2.** Concordance of VAF and alterations based on mutational profiles.
